# Group psychoeducation for persons with bipolar disorder in Rwanda: a study protocol for a randomized controlled trial

**DOI:** 10.1186/s13063-022-06926-1

**Published:** 2022-12-02

**Authors:** E. Musoni-Rwililiza, C. J. Arnbjerg, C. Murekatete, J. Carlsson, P. Kallestrup, D. Gishoma

**Affiliations:** 1 College of Medicine and Health Sciences, University of Rwanda, Kigali, Rwanda; 2 Mental Health Department, University Teaching Hospital of Kigali, Kigali, Rwanda; 3 Center for Global Health, Department of Public Health, Aarhus University, Aarhus, Denmark; 4 Competence Centre for Transcultural Psychiatry (CTP), Mental Health Centre Ballerup, Ballerup, Denmark; 5 Department of Clinical Medicine, University of Copenhagen, Copenhagen, Denmark

**Keywords:** Global mental health, Bipolar disorder, Psychosocial interventions, Psychoeducation, Low- and middle-income countries, Rwanda

## Abstract

**Background:**

The efficacy of psychoeducation as an add-on treatment to pharmacotherapy is well documented in treating symptoms and in relapse prevention for persons with bipolar disorder in western countries. Yet, no studies on psychosocial interventions for persons with bipolar disorder have been conducted in a low-income country in Africa.

**Aim:**

To develop a bipolar group psychoeducation program contextualized to the Rwandese setting, and determine its effect on symptom severity, medical adherence, and internalized stigma.

**Methods:**

A culturally adapted guide manual was developed by local mental health professionals, including nurses, psychologists, and medical doctors. In-depth interviews with participants were held prior to and will be held following the intervention to address the cultural aspect of living with bipolar disease and the impact of the program. A two-armed randomized controlled trial has been set up at the tertiary mental health hospitals in Rwanda, with an intervention and a waiting list arm. A sample size of at least 50 in each arm was calculated as a requirement. The study’s primary outcome measure will be the difference in relapse rate measured on the Young Mania Rating Scale and Hamilton Depression Scale-17. Differences in mean change on scales for medical adherence and internalized stigma will be secondary outcomes. Data will be analyzed according to the intention-to-treat principle. Participants will be assessed subsequently at baseline, at the end of the intervention period, and three months and 12 months post-intervention.

**Discussion:**

This study will be one of the first intervention trials on bipolar disorder in a low-income country. If proven successful in reducing morbidity and increasing the quality of life in persons with bipolar disorder, it is anticipated that the psychoeducation program can be implemented at the district and community level and act as inspiration for other low-resource settings.

**Trial registration:**

ClinicalTrials.gov NCT04671225. Registered on November 2020.

## Background

Mental health and neurological disorders constitute 13% of the global burden of disease [1]. Alarmingly, this burden has risen by 41% in the last 20 years [2]. In 2016, 18.5 million years were lost to disability due to mental health problems in the African region. In comparison, the disease burden of infectious and parasitic diseases in Africa was 13.1 million years lost to disability that same year [3]. Currently, three out of four people with mental health problems live in low- and middle-income countries (LMICs), where fewer than 1 in 50 people with severe mental disorders receive evidence-based treatment, and in most countries, less than 1% of the annual health budget is allocated to mental health [4–6]. The situation has been called both a failure of humanity and one of the most neglected global health issues of our time by some of the leading experts in the field of global health [7].

It is estimated that severe mental disorders (i.e., severe depression, bipolar disorders, schizophrenia, and other psychotic disorders) have a two to three times higher average mortality than the general population [8]. Treatment rates for these disorders are low in LMICs, where treatment gaps of more than 90% have been documented [9, 10]. This might explain why a considerable proportion — up to 50% — of individuals seeking care for mental disorders in Africa consult traditional and religious healers in their pathway to mental health care [11].

One of the major barriers in decreasing the treatment gap is the lack of human resources. In response to the shortage of health professionals, experts and WHO advocate that mental health care must be delegated to non-specialist health workers trained to deliver interventions for specific mental disorders [12, 13]. In Rwanda, a small Sub-Saharan country with more than 12 million citizens, the total number of medical doctors specialized in mental health is 12 in 2022. So far, no treatment guidelines on mental health disorders, including BD, exist in Rwanda.

Rwanda’s health care system is organized along with the principles of the WHO on primary health care [14, 15]. Health posts and health centers are the first points of contact for patients. These centers also coordinate all outreach and prevention activities held at the community level, carried out by Community Health Workers (CHWs). The next level of care is the 46 District and Provincial Hospitals, which has a mental health department staffed by a permanent team composed at least of a psychiatric nurse and/or a psychologist. At the top of the mental care system in Rwanda, there are four national referral hospitals offering outpatient care (Kigali Teaching Hospital - CHUK, Butare Teaching Hospital - CHUB, Rwanda Military Hospital, King Faisal Hospital) and two mental health hospitals providing both inpatient and outpatient care (The Neuropsychiatric Hospital of CAREAS-Ndera and the Huye Isange Rehabilitation Center).

Bipolar disorder (BD) is characterized by periodic episodes of elevated moods and depression, which co-occur with changes in activity or energy and is associated with cognitive, physical, and behavioral symptoms [16]. The efficacy of psychoeducation as an add-on treatment to pharmacotherapy is well documented in treating symptoms and in relapse prevention initiatives with respect to BD in western countries [17]. Psychoeducation is believed to empower the patient to take an active role in the therapeutic process, thereby potentially reducing stigma, guilt, and helplessness, improving medical adherence, and engaging caregivers [18]. Yet, no studies on psychosocial interventions for BD have been conducted in a low-income country in Africa [19].

In September 2014, the outpatient clinic of CHUK launched a psychoeducation program for BD. The manual for the program is entitled “Life Goals Program” (LGP) and was designed back in 1996 by Bauer and McBride [20]. LGP is a manual-based structured group psychotherapy program centered on behavioral principles from social education and self-regulation philosophies. Nevertheless, the effect of psychoeducation in Rwanda is unexplored, and psychoeducation is not part of the standard treatment.

Given the enormous shortage of skilled mental health professionals in Rwanda, the question that emerges is whether simple interventions such as psychoeducation could potentially be feasible and effective in a low-resource setting and potentially decentralized from the referral hospitals. Herein, we describe the protocol of a study designed to address gaps in the management of persons with BD in a low-income country in Africa. Currently, the study closed for recruitment, and the intervention is ongoing.

The primary aim of the study is to determine the effect, feasibility, and acceptability of psychoeducation for patients with BD in Rwanda.

## Methods

This trial protocol follows the guidelines for standard protocol development and reporting found in the Standard Protocol Items: Recommendations for Interventional Trials (SPIRIT) 2013 statement [21]. The completed SPIRIT Checklist for this study can be found in the Additional File.

## Trial design

**Fig. 1:**
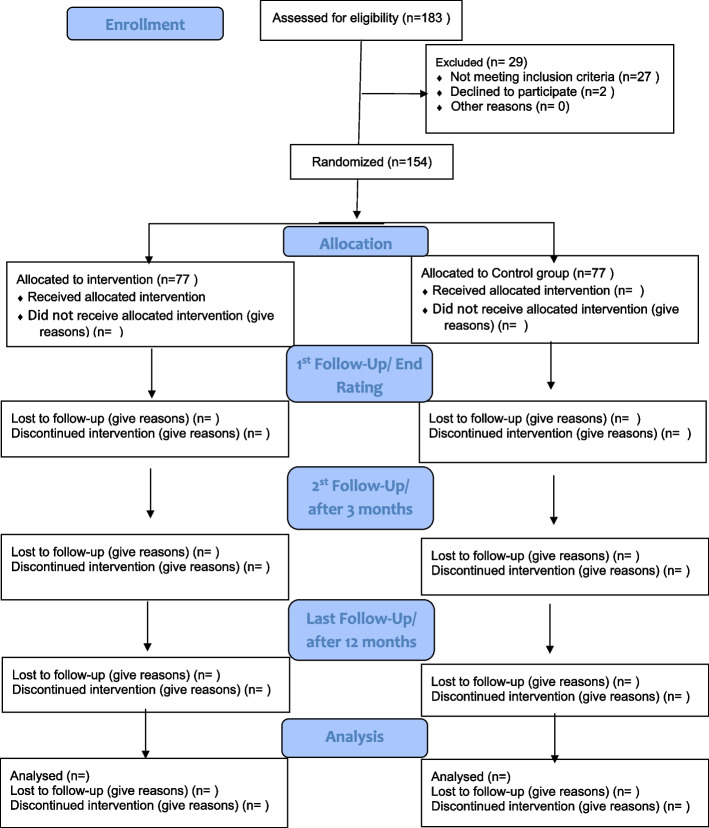
Planned flow chart of participants through the trial

The study is an open-label, two-arm randomized controlled trial with equal allocation between intervention and waiting list control arm. The psychoeducation intervention will comprise 8–12 sessions given over 12 weeks with assessments of outcomes at baseline, end-study, and at 3rd and 12th months follow-up of the study. An illustration of recruitment numbers, scheduled enrolment, interventions, and assessments is provided in Fig. 1.

### Study settings and participants

Participants diagnosed with BD type I or II were recruited from the two referral hospitals with mental health capabilities in Rwanda; CHUK and CARAES-Ndera Hospital.

CHUK is the largest referral hospital in Rwanda, located in the District of Nyarugenge, Kigali City. The hospital does not provide inpatient care for psychiatric patients, yet it is the second biggest outpatients’ mental health clinic besides CARAES-Ndera Hospital. The CHUK Mental Health Department offers a range of care and treatment for people experiencing common mental health disorders such as depression, bipolar disorders, psychotic disorders, panic and anxiety disorder, obsessive-compulsive disorder, and post-traumatic stress disorder (http://chuk.rw/about-chuk).

CARAES-Ndera Hospital is a mission health facility and the sole neuropsychiatric hospital in the country with inpatient care that offers specialized healthcare in psychiatry and neurology. The Hospital is located 17 kilometers from Kigali City, in Gasabo District (https://www.CARAES-Nderahospital.rw/1/overview).

### Inclusion criteria

- A diagnosis of BD type I or II that meets DSM-V given by a trained psychiatrist
- Adults (18 years or older)
- Signed informed consent provided

### Exclusion criteria

- Patients in a current depressive (above or equal to 17 on the Hamilton Depression Scale-17) or hypomanic- or manic phase (scores above or equal to 12 on the Young Mania Rating Scale)
- Previous participation in any structured psychological intervention, such as psychoeducation or cognitive remediation
- Mental retardation stated in medical records
- Current alcohol or drug-use disorder
- Insufficient understanding of Kinyarwanda
- Deafness
- Lack of informed consent

### Recruitment procedures

Clinical staff members at the two referral hospitals assisted in the recruitment of participants during their clinical work. Participants who had been in contact with the mental health department within the last year were prioritized.

### The intervention

Participants will be divided into groups of 8–10 members. Each psychoeducation group will have two facilitators, one psychologist, and one mental health nurse. The facilitators will get training on how to facilitate group psychoeducation through workshops and seminars.

With the permission already obtained from Professor Mark S. Bauer, the structured manual will be inspired by the Life Goal Program by Bauer et al. [20, 22] but will need cultural adaptation to the Rwandese setting. For further information regarding the Life Goal Program, please refer to www.lifegoalscc.com/overview, where a new modular form can be found and utilized, without charge.

Planned focus group discussions and in-depth interviews have been conducted and assisted the transcultural adaptation of the program, which in turn ensured the involvement of patients. The participants of the focus groups will be former participants and facilitators in the psychoeducation program from 2014. In-depth interviews will be held with naive psychoeducation participants focusing on the living experience of BD in Rwanda. A total of 8–12 sessions are expected to be included in the final manual. Following the finalization of the intervention, subsequent evaluation interviews will be conducted with study participants. The adapted manual utilized in the intervention will be made in the language Kinyarwanda and translated into English by a professional translator.

### Waiting list as the control group

As a control group, we will use the “Early intervention versus delayed intervention strategy”. The participants randomized to the waiting list will do their group psychoeducation 12 months after the intervention group; consequently, they will be used as the control group. Evaluations and assessments will be done in parallel with the intervention group at the screening day, three months after the group psychoeducation, and 12 months after the intervention. While waiting, the participants on the waiting list will receive the standard treatment care, which in Rwanda, mainly consists of taking prescribed medication from the outpatient clinic.

### Primary and secondary outcomes

All outcomes have been assessed at baseline and will be assessed at the first evaluation, which will take place immediately after 8–12 sessions of group psychoeducation (the intervention), then at the 3rd and 12th months follow-up. The outcomes are based on the most commonly used measurements for trials on psychoeducation for individuals with BD in high and middle-income countries [18, 19].

#### Baseline data

The Mini-International Neuropsychiatric Interview (MINI) [23] was applied to confirm the psychiatric diagnosis. Demographic information regarding the participants’ living circumstances, past psychiatric history, and the use of mental health service data was collected from interviews. Finally, past traumatic events and history of suicidal behaviors were recorded using Life Event Checklist for DSM-5 (LEC-5) [24] and The Columbia-Suicide Severity Rating Scale (C-SSRS) [25].

#### Primary outcomes

Relapse is defined as hospitalization or a new mood episode, such as mania when the participant scores above or equal to 20 on the Young Mania Rating Scale (YMRS) [26] or hypomania when the score is above or equal to 12 on the YMRS. For depressive episodes, the score should be above or equal to 17 on the Hamilton Depression Scale-17 (HDRS-17) [27], and for mixed episodes, the scores should be above or equal to 20 on the YMRS and equal or above 12 on the HDRS-17.

#### Secondary outcomes

**Fig. 2:**
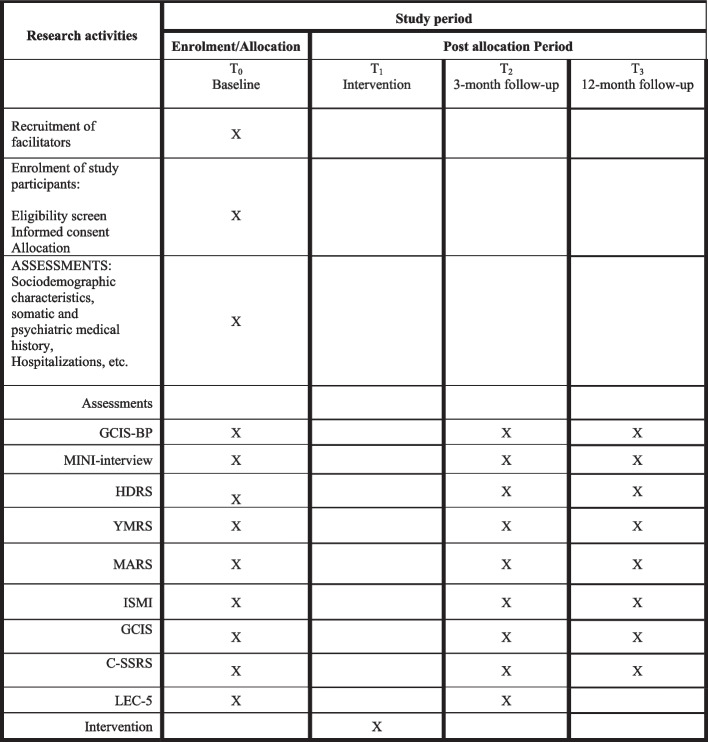
SPIRIT figure. Schedule of enrolments, intervention, and assessments

The shortened version of the Clinical Global Impression Scale for Bipolar disorder (CGI-BP) will be used to assess improvement in symptom severity observed by the clinicians [28]. The Medication Adherence Rating Scale (MARS) will be used to evaluate medical adherence [29]. All participants filled in a knowledge test and questions about self-stigma, Internalized-Stigma of Mental Illness Scale (ISMI) [30], and suicidal thoughts before and after the psychoeducation. An illustrative overview is shown in Fig. 2: schedule of enrolment, interventions, and assessments.

The only validated tools in Kinyarwanda are the MINI, LEC-5, and the HDRS. In parallel, data collection is ongoing in order to validate and culturally adapt the four remaining tools in Kinyarwanda.

### Sample size calculations

In the literature on group psychoeducation, 13 out of 18 RCTs have reduction in general psychiatric symptom severity, the incidence of relapse, and hospitalization as the main outcomes [18]. We reviewed 18 RCTs on group psychoeducation for BD before we selected a study by Colom et al. as the base of our power calculation [18, 31]. The incidence of relapse in the study was (92%) (55 subjects) in the control group vs. 67% (40 subjects) in the psychoeducation group. For our study, a sample size of 40 patients for each arm is required to achieve a level of 80% power with a 5% level of significance when comparing the mean change in each intervention with the control arm via a two-sample *t*-test. Adjusting for a drop-out rate of 20%, 40/(1−(20/100)), 50 participants will be needed for each group**.**

### Randomization and blinding

Randomization to either the intervention or the waiting list group followed immediately after baseline data collection. An independent statistician who is not involved in clinical assessments generated the randomization procedure. The allocation sequence was implemented using the REDCap (Research Electronic Data Capture) electronic system with a ratio of 1:1 and a block size of 4 and 6. When a patient was found eligible for inclusion by the clinical staff and signed the consent form, a third party who was not a part of the clinical staff at the hospital accessed the REDCap system, where the computer generated the allocation. Blinding of participants and clinicians is not possible due to the nature of the treatment intervention.

### Adherence to the study

Before each session, facilitators will be calling the participants to remind them about the next psychoeducation session.

### Data collection, management, and integrity

To ensure the safety and high quality of the data, the data collectors will receive intensive training on data collection and how to use the outcome tools.

All data will be entered and stored in the Electronic Data Capture system, REDCap. This system is compliant with the EU General Data Protection Regulation (GDPR) and Good Clinical Practice guidelines (GCP). The study adheres to all GDPR regulations. A trial ID will be assigned to all participants, and personal information will be stored securely and separately. Data will be entered directly into electronic Case Report Forms (CRF) using REDCap. Questionnaire data will be filled out manually and entered directly into REDCap by the research assistants and the investigators at baseline and follow-up. If access to the REDCap system is not possible, data will be collected on paper CRF and entered into REDCap when access has been restored. The research team will regularly conduct quality checks and verification of entered data at the aggregated and individual participant levels. These checks include the identification of missing data, internal consistency, the range for data values, and obvious errors. Once a data collection form has been checked and verified, it will be locked for further editing. The hard copy questionnaires will be stored in the field office accessible by the two principal investigators.

### Statistical analysis

All analyses will be carried out following the intention to treat principle, i.e., without regard to the compliance of individuals within their allocated study arm. Reasons for potential drop-outs will be accounted for and reported. Estimates of the effectiveness of the intervention and 95% confidence intervals will be adjusted for differences in baseline scores by analysis of covariance. Two-sided *p* values will be reported at a 95% confidence interval with a statistical significance level of less than 0.05. Continuous measures will be compared using t-tests, while binary outcomes will be analyzed using Chi-square or Fisher’s exact test if values are small. Multiple regression analysis will be performed to adjust for age, sex, and severity of illness. To check the robustness of the results, the primary outcome will also be analyzed using a per-protocol approach, including only participants who completed at least half of the psychoeducation sessions. Data analysis will be performed using STATA version 17 software (StataCorp, College Station, TX, USA).

### Ethical considerations

This study will be carried out in accordance with the Declaration of Helsinki. The study's ethical approvals have been granted by the University of Rwanda College of Medicine and Health Sciences-Institutional Ethical Review board and the CARAES-Ndera Hospital and University Teaching Hospital of Kigali-CHUK ethical review boards. Furthermore, the study investigators have obtained approval to carry out the research from the National Council for Sciences and Technology (NCST), as required for all researchers in Rwanda. Anonymity and confidentiality of participants will be ensured by assigning a study ID number to all participants. Informed consent will be obtained from all participants.

### Compensation

It is anticipated from the experience of conducting psychoeducation in Rwanda for patients with BD that the trial brings nearly no risk to the study participants; for this reason, the study is without an insurance plan. As Rwandan regulation requires, each study participant will receive a small compensation — 10.000 Rwandan Francs — to cover their transportation expenses from their villages to the research venue.

### Intervention fidelity

The fidelity of the study will be guaranteed, starting from the pre-intervention training of the facilitators. The facilitators will be explained the importance of enduring with all intervention constituents in the experimental arm. Moreover, the intervention will follow a structured guide manual that all facilitators will be trained in. Finally, participants’ attendance is registered after each psychoeducation session.

### Dissemination plan

The project aims to direct research and policy attention toward mental health globally and at national, and local levels. For the dissemination of the outcomes of the project, we will (a) publish articles in peer-reviewed scientific journals; (b) present results at international scientific meetings and conferences; (c) organize local meetings with key stakeholders during the full extent of the project; and (d) trial results will be communicated to participants in their routine clinical outpatient care.

The Vancouver rules for authorship will be followed. There will be no use of professional writers.

## Discussion and implications

This study protocol describes the first RCT to examine the effectiveness of a complex psychosocial intervention for persons with BD in a low-income country in Africa.

Facing the enormous treatment gap for mental health and the tremendous lack of human resources for health, there is an urgent need for evidence-based, operationally well-documented innovations that are compatible with the realities at a population level in resource-constrained settings. Only a very limited number of studies in LMICs have been conducted on severe mental disorders. This project is essentially this quest.

A limitation to the trial is that neither the patient nor the clinicians are blinded to treatment groups, and the outcome tools need to be validated before use.

Potential positive outcomes may be beneficial not just in Rwanda but also in similar low-resource settings elsewhere. If proven successful, relevant next steps will include having the newly established research group expand its focus area to severe mental health disorders other than BD; or conduct studies on the drug treatment of BD in low-income settings. Moreover, the project will serve to enhance local research capacity.

### Trial status

The trial has been closed to participant recruitment, but the study is ongoing. The trial recruitment and preparation of the trial materials began in February 2021.

The trial protocol has been submitted for publication after the end of recruitment due to a change in the original plan for recruitment due to the COVID-19-pandemic. Instead of recruiting consecutively over 12–18 months, we had to plan for a shorter inclusion period, as national lockdowns were imposed to contain the spread of the virus. Initially the intended demographic would be representative of the country as a whole; however, the inaccessible travel options, travel restrictions, and intermittent regional lockdowns due to COVID-19 necessitated a prioritization of participants living in and in the vicinity of the capital city of Kigali.

The study protocol was registered to clinicaltrials.gov before starting the baseline survey. The protocol version number is NCT04671225 and was entered in November 2020. The endpoint assessment of all the participants is expected to be completed by the end of September 2022.

## Data Availability

Not available.

## Abbreviations

AU: Aarhus University
BP: Bipolar disorder
CHUK: University Teaching Hospital of Kigali
CHWs: Community Health Workers
CARAES-Ndera: NeuroPsychiatric Hospital CARAES Ndera
CTP: Center for Transcultural Compentency
CMHS: College of Medicine and Health Sciences
DSM-V: Diagnostic and Statistical Manual-5^th^ Edition
GDPR: General Data Protection Regulation
GCP: Good Clinical Practice guidelines
HDRS-17: Hamilton Depression Scale-17
ISMI: Internalized-Stigma of Mental Illness Scale
LMICs: Low- and middle-income countries
LGP: Life Goals Program
LEC-5: Life Event Checklist for DSM-5
C-SSRS: The Columbia-Suicide Severity Rating Scale
CGI-BP: Clinical Global Impression Scale for Bipolar disorder
MINI: The Mini-International Neuropsychiatric Interview
MARS: The Medication Adherence Rating scale
NCST: National Council for sciences and technology
RCT: Randomized controlled trial
REDCap: Research Electronic Data Capture
SPIR IT: Standard Protocol Items: Recommendations for Interventional Trials
WHO: World Health Organization
YMRS: Young Mania Rating Scale
